# Plant Cell Cancer: May Natural Phenolic Compounds Prevent Onset and Development of Plant Cell Malignancy? A Literature Review

**DOI:** 10.3390/molecules21091104

**Published:** 2016-08-23

**Authors:** Hassan Rasouli, Mohammad Hosein Farzaei, Kamran Mansouri, Sara Mohammadzadeh, Reza Khodarahmi

**Affiliations:** 1 Medical Biology Research Center, Kermanshah University of Medical Sciences, Kermanshah 6714967346, Iran; h3n.rasouli@gmail.com (H.R.); mh.farzaei@gmail.com (M.H.F.); kamranmansouri@gmail.com (K.M.); sara3ms@yahoo.com (S.M.); 2 Pharmaceutical Sciences Research Center, Faculty of Pharmacy, Kermanshah University of Medical Sciences, Kermanshah 6714967346, Iran; 3 Nano Drug Delivery Research Center, Faculty of Pharmacy, Kermanshah University of Medical Sciences, Kermanshah 6714967346, Iran

**Keywords:** phenolic compounds, flavonoids, cancer, secondary metabolites

## Abstract

Phenolic compounds (PCs) are known as a chemically diverse category of secondary and reactive metabolites which are produced in plants via the shikimate-phenylpropanoid pathways. These compounds—ubiquitous in plants—are an essential part of the human diet, and are of considerable interest due to their antioxidant properties. Phenolic compounds are essential for plant functions, because they are involved in oxidative stress reactions, defensive systems, growth, and development. A large body of cellular and animal evidence carried out in recent decades has confirmed the anticancer role of PCs. Phytohormones—especially auxins and cytokinins—are key contributors to uncontrolled growth and tumor formation. Phenolic compounds can prevent plant growth by the endogenous regulation of auxin transport and enzymatic performance, resulting in the prevention of tumorigenesis. To conclude, polyphenols can reduce plant over-growth rate and the development of tumors in plant cells by regulating phytohormones. Future mechanistic studies are necessary to reveal intracellular transcription and transduction agents associated with the preventive role of phenolics versus plant pathological malignancy cascades.

## 1. Introduction

Phenolic compounds (PCs) are secondary metabolites ubiquitously present in the plant kingdom [1,2,3,4,5,6,7,8,9,10,11,12,13]. PCs have characteristic aromatic rings with some hydroxyl groups affixed [8,11,14,15,16]. Recently, researchers have suggested that secondary metabolites are not only extended commodities of primary metabolism, but that they also contribute to plants’ survival in the environment [8,11,14,15,16]. Mounting evidence suggests that PCs are responsible for the beneficial effects of natural agents, and their biological activity has been investigated in various in vitro and in vivo models [6,8,16,17]. It has been found that PCs possess a wide range of biological activities, including antioxidant properties [11,12,14], fighting against free radicals [18,19], chelating metal ions [9,18], antimicrobial activity [7,9,20], anticancer characteristics [19,21], anti-inflammatory activity [6,8,10,16,19], etc.—indicating the crucial role of PCs for the maintenance of human health [1,2,3,4,5,6,7,8,9,10,11,12,14,15,16,17,18,19,20,21]. In recent years, the food industry has applied them for the improvement of food quality [9]. Several PCs, such as hydroxybenzoic acids, catechins, and curcuminoids have major preventive impacts on the growth of cancer cells [22]. Regarding the ubiquitous distribution of PCs in fruits, vegetables, and cereal grains, their presence in a balanced, healthy diet can inhibit the progression of some types of cancers [6,11,17]. Some reports indicate that oxidative stress is a critical factor for cell damage [23]. Reactive oxygen species (ROS) and reactive nitrogen species (RNS) may be generated in oxidative stress reactions [24,25]. Subsequent to the interaction of plant cells with oxidation factors, antioxidant substances (i.e., PCs) are produced, which react with ROS and free radicals [11]. PCs can protect the cells against any chemical injuries, such as those caused by free radicals [26]. There are some reports showing an increase in ROS in cancer cells compared to healthy cells [3,14,21,22]. A decrease in the concentration of antioxidants has a pivotal role in the generation of ROS and other oxidative products, resulting in the destruction of whole cell systems [3,7]. This condition occurs in different pathological conditions, such as in malignancies [21]. The aim of the current study is to highlight the role of PCs in plant defensive mechanisms against pathological conditions such as malignancies.

## 2. Structure and Synthesis

### 2.1. Classification of PCs

PCs encompass a wide range of chemical structures, with a variety extending to the presence of polymerization and substitutions of the PC basic skeleton, along with the degree of oxidation [11,12,14,15,16,19,20,21,25,26]. In recent years, phytochemicals have been categorized as fundamental or secondary factors, depending on their positions in plant metabolism [27]. Essential factors include the ordinary sugars, amino acids, proteins, purines, and pyrimidines of nucleic acids, chlorophylls, etc. [28]. Secondary elements are non-essential plant chemical substances; for example, plant steroids, terpenes, alkaloids, flavonoids, lignans, saponins, curcuminoids, phenolics, and glucosides [7,9,11,12,20,25,28]. Previous studies have indicated that PCs include abundant and structurally broad-spectrum phytochemicals [27]. PCs can be divided in to four main groups: (1) Phenolic acids, (2) Flavonoids, (3) Tannins, and (4) Stilbenes (Table 1) [26].

### 2.2. Production of PCs

The biosynthetic pathway of PCs is well known [9]. Although a wide variety of phenolic compounds are present in plants, most of them are generated by aromatic amino acids, including phenylalanine, tyrosine, and tryptophan [8,12,23,29]. Aromatic amino acids are the primary components in the synthesis of polyphenols [7]. Phenylpropanoids and shikimic acid routes are the main pathways in the generation of these ubiquitous compounds [13,30]. Flavonoids are also able to be synthesized from the malonic acid route, but its role in the biosynthesis of PCs is rather minimal [29]. The enzyme phenylalanine ammonia-lyase (PAL) (EC 4.3.1.5) has a major role in the biosynthesis of these ubiquitous compounds [7,13,31]. Inhibition of the PAL enzyme reduces the formation of these compounds [31]. Including the role of PAL in the production of phenolic compounds, it seems that the factors associated with the regulation and control of the quality and quantity of phenols in plant tissues still remain controversial. Much of this controversy arises from the many factors involved in the interaction between genotype and environment. This has led to a wide variation in plant phenol production among and within species over time [13].

### 2.3. Storage Sites of PCs

The synthesis of PCs happens in the chloroplast, the endoplasmic reticulum membrane, and the cytoplasm [32]. PCs are normally placed in the vacuoles of the epidermal cells, guard cells, and sub-epidermal cells of leaves, the aerial regions of monocotyledonous and dicotyledonous plants [30], cortex parenchyma cells, vascular parenchyma cells, and cell walls [33]. The accumulation of PCs in a specific tissue reflects its biological ability or indicates its participation in plant-environment interactions [33,34]. These compounds are also stored in the gymnosperm of the rhizome [34]. The concentration of a specific phenolic compound within a plant tissue is dependent on the season, and may also vary at different stages of growth and development [35]. Several internal and external factors—including trauma, wounding, drought, and pathogen attack—affect the synthesis and accumulation of PCs [30,35,36].

## 3. Role of PCs in Plant Defense Mechanisms

### 3.1. Plant-Environment Interactions and Functions of PCs

Ubiquitous PCs have critical functions in plant-environment interactions [37]. Plants are constantly exposed to attacks by pathogens, insects, and herbivores [38]. When a pathogen attacks a plant, in certain conditions a component of the pathogen may infect the plant [37,38,39]. To cope with the pathogen, the infected plant applies a rapid and strong defensive response, called the hypersensitive response (HR) [39].

HR is a rapid defensive mechanism against any stressful state in plants, including biotic and abiotic stimuli [40]. In this mechanism, highly localized cell death may occur [38,40]. Similar to animals, programmed cell death is a necessary mechanism for growth, development, and defense against diseases in plants [40,41,42]. When a plant is exposed to UV radiation [43], chemical toxins [44] and hypoxia [45] are arisen in the damaged cells [44,46], the involved tissue responds to these environmental stresses, actively. In the HR, plant cells die, and their cell walls deposit PCs to strengthen themselves [37]. When HR is activated inside the plant, structural alterations occur in plant cells that are submitted to stress (for example, a biotic stress) [40,47]. These alterations will immobilize the cytoplasm, separate protoplasts, and break down DNA [46,47]. In the end, this process is capable of destroying infected tissues, and necrosis may be observed on the surface of plants, thus preventing plants from becoming cancerous [46].

If the plant cannot limit the spread of pathogens, it is susceptible; if the opposite is true, it will be resistant [48]. Plant responses to pathogens or other stressful conditions create a defense reaction [37,48]. During HR in plants, PCs are placed in the infected tissues, and their chemical structure is altered; for example, the accumulation of PCs in injured tomato tissues [49] and the deposition of lignin polymers [50,51]. For the restriction of pathogens in local sites, cells adjacent to the injured tissues are triggered and increase the synthesis of PCs [52]. Some plants respond to pathogen attacks by accumulating phytoalexins, such as hydroxycoumarins and hydroxycinnamate conjugates [35,53]. On the other hand, when *Botrytis cinerea* (a fungal pathogen) attacks grapevine berries, an accumulation of stilbene (resveratrol) is observed in the infected region [54].

Abiotic tensions can also stimulate the production of PCs in plants [55]. Several investigations have shown that temperature may be positively or negatively related to the accumulation of PCs in vascular plants [55]. Xu and colleagues documented that temperature and light elevate PCs in winter berries when compared to summer berries [56]. Several studies observed that when HR occurs in plants, the injured cells became brown, suggesting the biosynthesis of PCs in the affected region [37,56]. PCs influence membrane perturbation, which is followed by a cascade of physiological effects that include improvement of plant-water relationships, stomatal function, and the rate of photosynthesis and respiration [13]. During HR in maize, it has been shown that the *lls1* gene encodes an aromatic ring-hydroxylating enzyme that is an intermediate factor in cell death in plants [48]. In general, most known effects of polyphenols on pathogens are negative [12]. Some reports show that when a plant is infected by fungal pathogens and HR is activated, the total amount of PCs is increased (Table 2). The synthesis, release, and accumulation of phenolics are central to many defense strategies employed by plants against microbial invaders [35]. Phenolics are synthesized when plant pattern recognition receptors recognize potential pathogens via the conserved pathogen-associated molecular patterns (PAMPs), leading to PAMP-triggered immunity [35,53]. As a result, the progress of the infection is restricted long before the pathogen gains complete hold of the plant [53,57].

Another example of PCs is lignin, which is incorporated in plants’ response mechanisms [71]. Lignin is a phenolic polymer which plays a critical role in solute conductance, mechanical support, and disease resistance [59,72]. In response to abiotic stress, injuries, or pathogenic infection, the deposition of lignins, lignin polymers, and other phenolic substances associated with the cell wall are seen [72].

Lignin not only acts as a physical barrier against pathogenic invasion [72], but it also decreases the diffusion of enzymes and toxins released by the pathogen to facilitate host tissue colonization [73]. Lignin also restricts the access of pathogens to plant water and nutrients essential to their proliferation [72,73]. There are other mechanisms showing the activities of PCs in the prevention of tissue injuries—e.g., dityrosine-mediated cell wall cross-linking [59].

When these responses happen in a rapid and coordinated way, they result in resistance against pathogens [74]. Resistance or susceptibility in the plant’s defense responses differ qualitatively and quantitatively, depending on leaf age, type of inoculation (single or multiple), and on the interactions between the plant and the pathogen [48].

### 3.2. Antioxidant Properties of PCs in Plants

Antioxidants are substances that can prevent the oxidation of oxidizable substances by quenching free radicals and reducing oxidative stress [75]. Reactive oxygen species are produced by living organisms as a result of normal cellular metabolism and environmental factors, such as oxidative stress [76]. ROS are highly reactive molecules and can damage cell structures such as carbohydrates, nucleic acids, lipids, and proteins and alter their function [77]. Oxidative stress is considered to be a basic factor in the increase of persistent degenerative ailments, such as coronary heart disorders, cancer, and aging [11,22]. PCs act as free radical acceptors and chain breakers [14,78]. They interfere with the oxidation of lipids and other molecules via the speedy donation of a hydrogen atom to radicals (R) [78,79]:

R + POH → RH + PO•(1)

The phenoxyl radical (PO•) is reasonably stable, due to resonance; for the same reason, a new chain reaction is not easily started [79,80]. Moreover, the PO• act as propagation terminators by reacting with different free radicals [78,79]:

PO• + R• → POR(2)

In plants, ROS substances are very dangerous for cells, and scavenge host enzyme systems and non-enzymatic antioxidants [80,81]. In plants, non-enzymatic detoxification procedures include morphological features such as waxy surfaces and leaf or chloroplast movement, non-photochemical quenching processes by various compounds (e.g., the violaxanthin-zeaxanthin cycle), and photorespiration [81,82]. Non-enzymatic antioxidants include flavonones, anthocyanins, α-tocopherol, ascorbate glutathione, carotenoids, phenolics, and polyols [80]. Botanical sources of these antioxidants not only play important roles in plant stress adaptation, but also retard aging and diseases related to oxidative damage in animals [83].

### 3.3. Role of PCs in HR

Some studies have indicated that during HR activity, some enzymes—such as PAL, polyphenol oxidase (PPO), and peroxidase (POX)—are elevated [84,85]. As described above, PAL is a main enzyme involved in the production of PCs [7]. In plant–pathogen interactions, phenol converts to lignins via the POX enzyme [86]. Thus, the accumulation of PCs and their oxidation through enhanced PPO and POX activity could be related to plant protection [87].

It is well documented that H_2_O_2_ is a signal molecule during the HR [88]. The HR is accompanied by a set of defense reactions, including the activation of defense genes [88]—especially genes encoding pathogenesis-related proteins [88]. The death of infected and stressed cells in order to prevent the systemic spread of a pathogen appears to be a conserved strategy in both plants and animals, and recent studies indicate that HR cell death could be considered programmed cell death (PCD), in which a limited number of cells die at the site of infection [39].

In some systems, H_2_O_2_ has been characterized as a diffusible PCD-mediating signal during HR [89], in which it is associated with a systemic signaling network, giving rise to HR in leaves of plants injured by virulent bacteria [90]. However, recent investigations of transgenic catalase- and peroxidase-deficient tobacco (i.e., in which endogenous H_2_O_2_ will not be readily catabolized) indicated that such plants have a hypersensitivity responsive to pathogenic injuries [89,90], therefore providing a direct role for H_2_O_2_ in HR cell death [89,90]. It has been shown that H_2_O_2_ induces PCD in soybean and *Arabidopsis thaliana* cell cultures [91]; however, recent evidence also suggests that cell death and the induction of defense genes are activated by the same signal but are regulated by separate mechanisms [91]. It is clear that both H_2_O_2_ and nitric oxide (NO) may mediate the transcription of specific genes; however, the mechanism by which this process occurs is not yet clarified [92]. It may be mediated by the activation of transcription factors through a phosphorylation cascade similar to the mitogen-activated protein kinase (MAPK) cascade [93].

### 3.4. Plant-Plant Interactions and the Accumulation of PCs

Allelopathy is defined as any direct or indirect effect of one plant on another that is mediated by the production of chemical compounds released into the environment [94]. Commonly, this term is most used to describe the chemical interaction between two plants [95,96]. In plants, allelochemicals can cooperate in leaves, bark, roots, root exudates, flowers, and fruits [96,97,98]. The delivery of allelochemicals into the rhizosphere is often thought to happen via leaching from leaves and other aerial plant regions, by volatile emissions, through root exudation, and by the breakdown of bark and leaf litter [96,98]. Phenolics have been classified by some as allelochemicals since the beginning of allelopathy studies [99,100]. In fact, many references about the physiological effects of phenolics exist, both alone and in combination with others. Phenolics have been shown to affect photosynthesis, respiration, water relationships, germination, growth, development, and many other physiological parameters [100]. Depending on the concentration, effects can be positive or negative for the plant receiving the phenolics, although some authors highlight the phytotoxicity of these molecules [96,99]. A list of the most relevant allelochemical compounds is displayed in Table 3.

**Figure 1:**
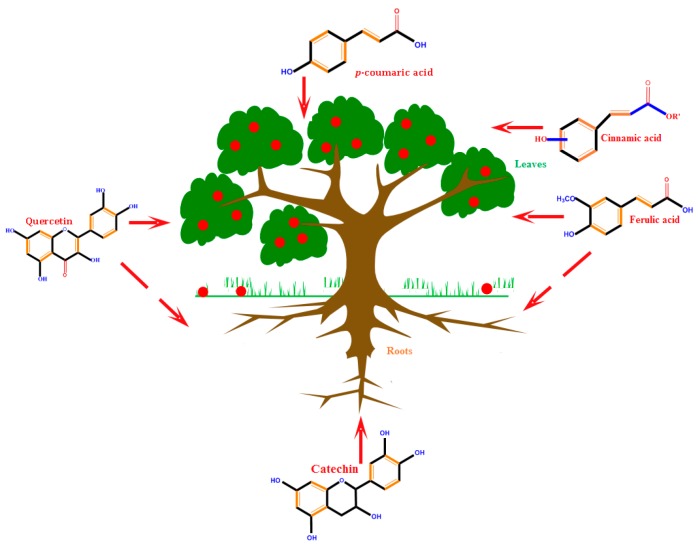
Distribution of the most relevant allelochemicals in the plant.

Allelochemical compounds are found in all plant parts. Figure 1 shows the most relevant release procedures in plants.

Phenolic compounds are a very significant group of allelochemicals, and most of them have inhibitory effects on the seed germination and seedling growth parameters of plants [104]. Low-molecular-weight phenolic compounds—released by plants and soil microbes—also represent an important group of compounds having major ecological functions [105,106]. However, many PCs containing monomers and polymers are allelopathically important, and may pose a serious threat to forest renewal [106]. However, little information is available as to what chemical properties of soils are affected by phenolic compounds, and how they influence soil processes [105].

## 4. PCs and Plant Tumors

### 4.1. Anti-Tumor Effect of PCs

Nowadays, the development of phytotherapies aiming at the inhibition of angiogenesis, in combination with classical anti-cancer therapies, is among the most intensively studied approaches for the treatment of cancer [107]. In vivo and in vitro studies have documented that PCs (especially flavonoid families) have anticancer activities [107]. Flavonoids quench ROS, induce apoptosis or caspase activities, prevent the proliferation of cancer cells and/or cyclin-dependent kinase (CDK) activities, and also block different cell cycles from cell lines [22]. Quercetin has been shown to reduce cell proliferation, cause cell cycle arrest in the G_0_/G_1_ phase, the G_2_/M-phase, and the S-phase, and induce caspase-3 activity and apoptosis in a concentration range of 10–100 μmol/L in in vitro experiments with various cell lines [108]. Resveratrol decreased proliferation and induced apoptosis and cell cycle arrest in the S-phase or G_2_/M phase [108].

Since these compounds are present in vegetables and some other foods [20], they may thus be used in treatment or as complementary agents in cancer treatment [22]. Today, interestingly, phytotherapy programs are being used alongside classical cancer treatments for cancer therapy [16]. Table 4 presents a comprehensive list of PCs and their mechanisms in the prevention of cancer cell line activity.

### 4.2. Tumor in Plant Cells

Cancer is identified as unconquerable cell growth and attainment of metastatic potentiality [3,21,22,107,112,115,118,119,121,124]. In animals, tumorigenesis refers to a process wherein a normal cell enters into uncontrolled division [22,107]. Tumorigenesis is a multi-step process characterized by the deregulation of various vital cellular functions, including proliferation, cell motility, adhesion, immortality, as well as proteolytic activity [22,107,112,115,117].

Neoplasms or tumors can develop in plant organs, wherein the plant cells divide uncontrollably to form hard outgrowths [125]. By far the most common example of this is crown gall disease, which is caused by *Agrobacterium tumefaciens* [126]. Agrobacterium is able to inject a piece of its genetic material into a plant’s genome, and this piece of bacterial genetic material carries genes that code for growth factors [126] which can cause the out-of-control growth of plant cells [125,126]. However, the formation of tumors in plants may be caused by several factors, including environmental (such as bacteria, viruses, insects, and worms) and genetic reasons [127]. Several studies have reported that genetic tumors occurred in *Melitotus alba* Desr, *Raphanun sativus, Pisum sativum* L., and other species [127]. The habituation of a plant’s callus to hormones in in vitro cultures may be attributed to genetic tumors [127]. This occurrence was first shown by Gautheret in 1942 for carrot cultivars growing in vitro in the absence of exogenous auxin [127]. Later, auxin habituation was described for *Nicotiana tabacum, Vitis vinifera, Helianthus annus, Lolium longiflorum, Glycine max, and Zea mays* [127]. In addition to auxin habituation, cytokinin habituation was shown in several studies [128]. Today, mounting evidence suggests that plant hormones have an essential role in uncontrolled growth and tumor formation [128]. Among phytohormones, auxins are a specific contributor to tumor formation [127]. An increase in auxin levels in a *N. glauca* × *N. langsdorfii* hybrid, as compared to wild-type species, caused the formation of a small tumorous structure [127]. Further, there was a positive correlation between auxin concentration and the tumor formation rate and size [127]. Matveeva and co-workers demonstrated that sensitivity to auxin in plant cells can lead to tumor initiation [127]. Cytokinins also play a crucial role in tumor induction [128]. For example, in maize, cytokinins accumulate in leaves infected by the basidiomycete fungus *Ustilago maydis*, leading to common corn smut-characterized by the production of tumors in susceptible aboveground plant tissues [128]. In general, it is thought that auxins and other hormones may have specific activity in different tissues at different time points during the development of tumors [127].

### 4.3. Roles of PCs during Plant Growth and Plant Tissue Culture

PCs are internal physiological modulators or chemical messengers within the intact plant [129]. Natural growth inhibitors are regulating substances which retard processes such as root and stem elongation, seed germination, and bud opening [130]. Many PCs inhibit the growth of plants or plant tissues which produce them [130,131]. Moreover, as mentioned in Section 3.4, some phenolics inhibit the growth of other plants or plant seeds when released into the environment [30,131]. Among the physiological responses of plants to phenolic compounds, the effect on energy metabolism—including respiration and oxidative phosphorylation—has been studied in only a few instances [130,131]. Table 5 presents the importance of the most relevant PCs to plant growth.

**Figure 2:**
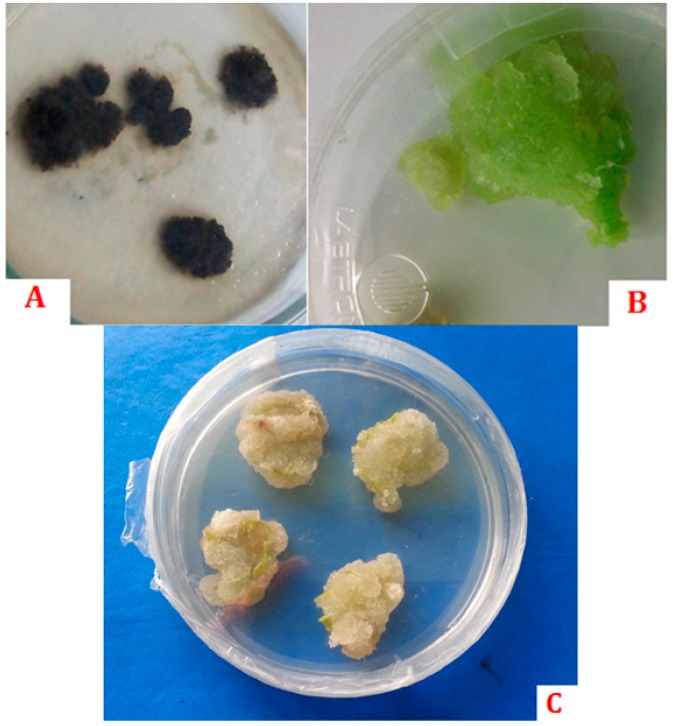
(**A**) Lethal browning effect of PC exudation; (**B**) Normal callus growth (in presence of ascorbic acid); (**C**) Normal wheat callus (in presence of PVP) [145].

Plant tissue culture is known as the science or art of growing plant cells, tissues, or organs on artificial media by separating them from the mother plant [132]. Plant tissue culture techniques can complete new plants from different explants by direct or indirect morphogenesis and by somatic embryogenesis [133,134]. It is a replacement plant propagation technique, and is being applied widely for the commercial propagation of a number of plant species, such as some medicinal plants [135]. The oxidation of exuded PCs causes darkening or browning of media, which prevents the intake of nutrients and ultimately causes the death of explants [134]. Their exudation is minimized through the application of different absorbents and antioxidants (Figure 2).

Table 6 summarizes the use of phenolic compounds in plant tissue culture. Mounting evidence suggests that phenolic compounds are unstable in media and that they may destroy explants [134]. Thus, for the prevention of killing effects (i.e., browning), it is necessary that antioxidant compounds (activated charcoal [134], PVP [132], ascorbic acid [135], citric acid [134], l-cysteine [134], or mercaptoethanol [134]) be added to media.

### 4.4. PCs as Inhibitor of Seed Germination

Phenolic compounds have a crucial role in plant seed germination [101,146]. Their presence and accumulation in soil can reach a threshold level, preventing pre-emergence seed germination or post-germination, growth, and other plant roles [139]. Different phenolic contents have been shown to prevent seed germination and seedling growth in plants (Table 5).

It has been documented that the prevention of seed germination in fruit was not generally related to a single component, but was due to the synergistic function of several components [147,148]. Some phenolic components existed in both seed coats and embryos that influence seed germination and dormancy [147]. Hydroxycinnamic acids, coumarins, tannins, and ferulic acid have been some of the usual preventers of seed germination [149]. It has been shown that phenolics can be active as germination preventers by preventing the transport of amino acids and the synthesis of proteins in seeds [148,149].

Another possible role of phenolic acids in seed germination can be their function in the production and decomposition of indoleacetic acid (IAA) [148]. In peach seeds, products of amygdalin decomposition (mandelonitrile, benzaldehyde, and cyanide) do not seem directly associated with the breaking of peach seed dormancy [148]. Bewley and Black [149] showed that the testa of the seed protects the embryo, and contains some phenolics. There are some external applications of PCs for seed germination as well [149]. In addition, some flavonoids are also able to inhibit coleoptile section elongation, stems and bud opening, and seed germination [130].

### 4.5. Do PCs Suppress Tumors in Plants?

The growth and development of plants is conducted by chemical substances (i.e., hormones) [146]. Plant growth regulators (or hormones) are small organic molecules that act inside plant cells and alter the growth and development of plants [150]. There are five groups of hormones which are involved in the growth and development of plants by different mechanisms; these include auxins, cytokinins, gibberellins, abscisic acid, and ethylene [151]. Growth promoters are involved in cell division, cell enlargement, pattern formation, tropic growth, flowering, fruiting, and seed formation [150]. Growth is defined as an irreversible increase in cell size along with protoplasm increase, which includes cell division and elongation [152]. Development is known as the form-changing of cells derived from zygotes or individual cells to cells with different biochemical and biophysical properties, having the same genetic contents [151]. Morphogenesis is the process wherein the origin of morphological characteristics and the main form of the cell takes shape [152].

Cytokinins and auxins seem to have a greater impact on the growth and development of plants [134,151]. Auxin is one of the most known hormonal plant growth regulators (identified by Charles Darwin’s experiments in 1880 and later coming to be known as a plant growth regulator [134]), and is characterized by its ability to induce cell elongation in stems and leaves and to increase photosynthetic activities in plants [150,153]. Auxin acts in the morphogenesis of cells by loosening the primary cell wall [153]. It increases the flexibility of the coleoptile cell wall, as well as the young and developing parts of the stem [151]. Cytokinins have been shown to participate in the regulation of numerous aspects of plant development—including the initiation of buds, flowering, abscission, and yield—by enhancing cell expansion [154].

**Figure 3:**
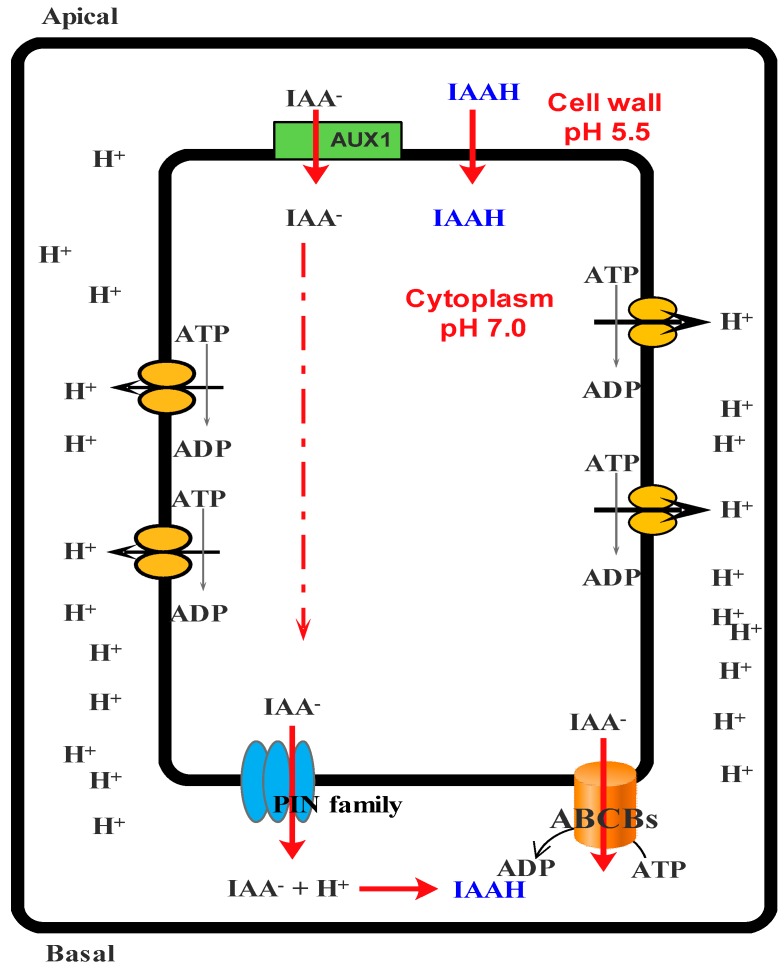
Simplified model for the entrance auxin into cell and response of cell to it. ABCB: ATP-binding cassette subfamily B; PIN: PIN-formed protein; AUX1: Auxin transporter protein 1.

The cell wall is the most important restricting factor for plant growth; thus, the action of auxin on the cell wall can result in the increase of cell size and growth [155]. The cell pumps protons into the cell wall environment in response to auxin, resulting in a decrease of pH and activation of cell wall-loosening enzymes (i.e., endoglucanases, pectinases, and xyloglucan endotransglycosylase) which subsequently lead to cell development (Figure 3) [156].

**Figure 4:**
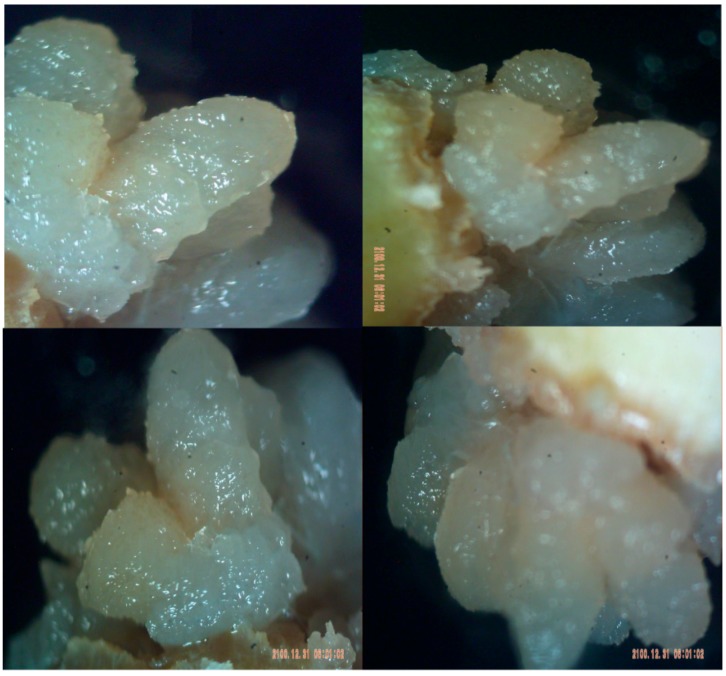
Callus structure and its similarity to tumor cells [145].

Petrasek and Friml determined that auxin has an important role in embryogenesis, root and shoot development, tissue development, and tropisms (phototropism, gravitropism) [156]. Under controlled conditions (i.e., in vitro plant tissue culture), it is possible to have the creation of tumor-like unorganized cell masses (or callus) by the regulation of auxin and cytokinin levels and callus induction [157] (Figure 4). Moreno et al. accomplished this using MS medium supplemented with NAA (0.5 mg/L) + 2,4-D (0.5 mg/L) + BA (2.0 mg/L) and culture at 25 °C for at least 2 weeks [158]. Additionally, through optimum growth media and the removal of morphogenesis ability, callus can be sustained for longer times [159].

**Figure 5:**
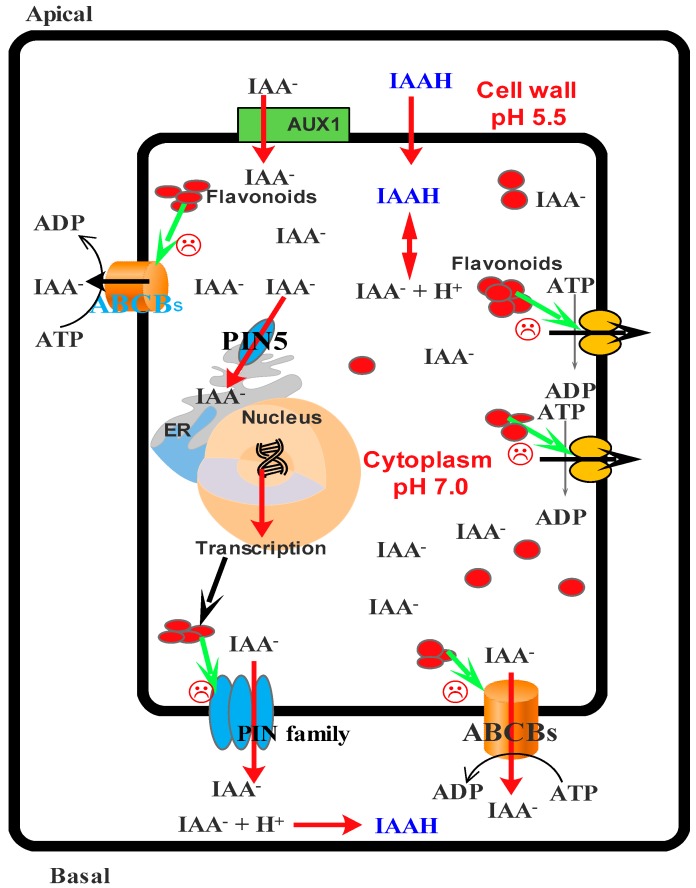
Prevention of auxin transport by PCs.

Auxin transportation by PIN families (PIN1-7), ABCBs1, 4, 19, and auxin transporter protein 1 (AUX1)/auxin transporter-like protein (LAX) provides growth and development by two routes: the long route (via mature phloem) and the short route (via vascular cambium) [160]. It is well-known that plant development is performed by the distribution of auxin in different tissues [160]. Auxin efflux transporters (e.g., ABCB1, ABCB19, and ABCB4) are directly inhibited by aglycone flavonols [161]. PCs inhibit auxin transference through protein phosphorylation, protein-protein interaction, and the prevention of ATPase activity or allosteric binding to them [162]. It is also suggested that these compounds are capable of inhibiting PIN transporters (Figure 5) [156,161]. Santelia and colleagues showed that PCs, in addition to preventing efflux transport of auxin, are also capable of inhibiting the polar transport of auxin [161].

**Figure 6:**
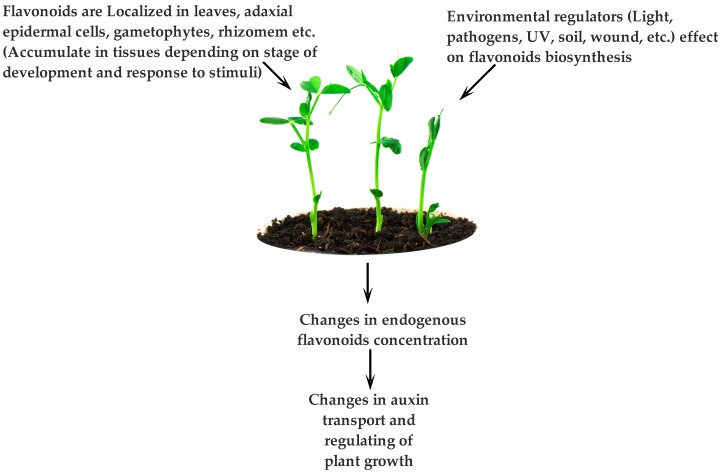
PCs (e.g., flavonoids) as endogenous regulators of auxin concentration.

PCs are endogenous plant compounds that are able to negatively regulate auxin transport and set tropic responses [163,164,165,166]. Environmental regulators (i.e., light, UV irradiation [167], pathogenic factors [168], soil type, and irrigation type [169]) affect PC biosynthesis [164,165,169,170]. A change in endogenous PCs is paralleled with a change in the transport of auxin hormone. Regarding the positive correlation between sites of PC accumulation (i.e., leaves and fresh parts of the plant) and auxin hormone (fresh tissues and meristematic regions), it is reasonable to conclude that PCs act as endogenous regulators of auxin hormone (Figure 6) [163,164,165].

Jacobs and Rubery showed that some PCs (e.g., quercetin, apigenin, and kaempferol) are able to inhibit auxin transport in vitro [171]. Brown et al. confirmed previous findings [171], and also showed that under in vivo conditions in *Arabidopsis*, endogenous phenolics regulate auxin transporting in various tissues [164].

Red light stimulates kaempferol synthesis (an enzyme co-factor of IAA oxidase), and also promotes quercetin synthesis (the inhibitor of IAA enzyme in leaves) [151,152]. However, it is worth note that when auxin transport sites are inhibited by PCs, additional IAA does not affect plant growth. Considering PCs in young and fresh parts of the plant and the presence of auxin in the same areas, it seems that these compounds control minimum auxin concentration and prevent over-growth of cells [151,152,172,173].

Environmental stress increases the activity of the peroxidase III enzyme, which may induce a morphogenic response to stress and also regulates auxin concentration in special tissues via the antioxidant activity of some PCs (i.e., quercetin) [173,174].

As demonstrated in Figure 5, PIN5 is the only protein present in the endoplasmic reticulum (flavonoids synthesis site), and its presence confirms the role of PCs as internal regulators of cell growth [156]. In general, these compounds depress the growth of plant sections and act as antagonists to plant hormones such as auxin, gibberellin, and cytokinin [130]. These effects on plant growth are well described in many studies [130,163,166].

## 5. Concluding Remarks

PCs are a major biologically and chemically diverse category of secondary metabolites with a remarkable physiological role in plant metabolism and resistance. Additionally, regarding their numerous biological effects in the prevention and treatment of illnesses, a large body of evidence supports the beneficial health properties of dietary polyphenols in humans. In addition to their antioxidant action, these compounds are capable of inhibiting plant growth by regulating the transport of phytohormones in plant tissues. A large body of cellular and animal evidence carried out in recent decades has confirmed the anticancer role of PCs. The accumulation and distribution of PCs can affect plant growth. Under normal conditions, it seems that PCs may prevent the over-growth of plants by the endogenous regulation of auxin transport, resulting in the prevention of cell wall development, and subsequently the prevention of tumorigenesis.

Regarding the key role of cell division in cell growth, the role of auxin and cytokinins in cell division, and the over-sensitivity of plant cells to auxin hormone, it can be concluded that PCs regulate the auxin concentration gradient (by inhibiting polar transport) and local auxin concentration (through inhibition of IAA oxidase in tissues), resulting in growth delay. Thus, PCs can reduce growth rate and the development of tumors in plant cells by regulating phytohormones. It is suggested that future study focus on perfect intracellular mechanisms of PCs in the prevention of plant tumorigenesis.

## Figures and Tables

**Table 1:** Main classes of phenolic compounds (PCs).

Polyphenols	Basic structure	Examples		
Phenolic acids	Hydroxybenzoic acids	Vanillic acid	Gallic acid	Syringic acid
	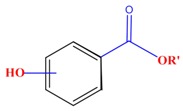	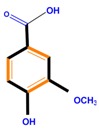	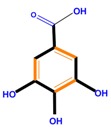	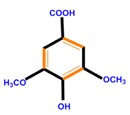
	Hydroxycinnamic acids	Caffeic acid	Ferulic acid	*p*-Coumaric acid
	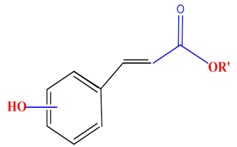	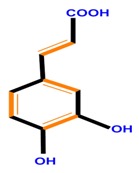	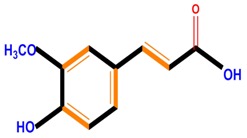	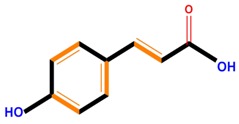
Flavonoids	Flavones	Chrysin	Luteolin	Apigenin
	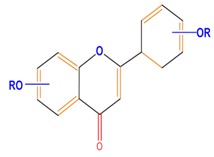	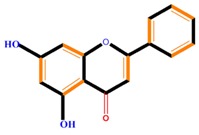	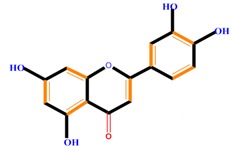	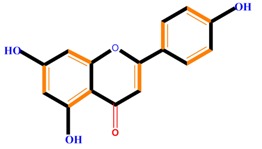
Flavonoids	Flavonols	Galangin	Kaempferol	Quercetin
	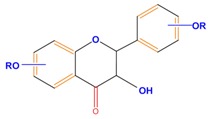	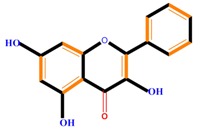	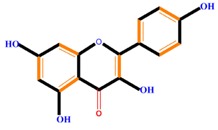	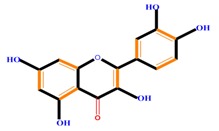
	Flavanones	Naringenin	Hesperetin	Eriodictyol
	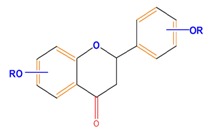	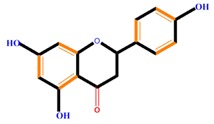	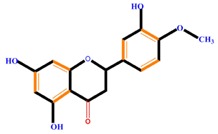	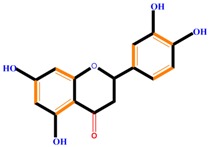
	Flavan-3-ols	Catechin	Epicatechin	Epigallocatechin (EGC)
	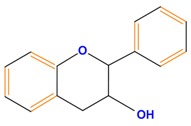	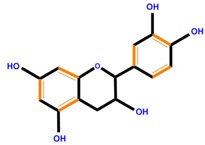	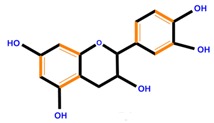	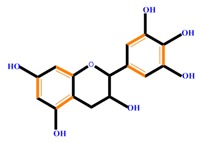
	Isoflavones	Genistein	Daidzein	Neobavaisoflavone
	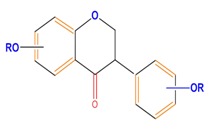	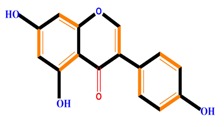	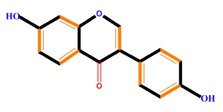	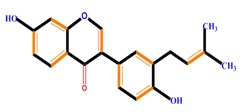
Flavonoids	Anthocyanidins	Cyanidin	Delphinidin	Pelargonidin
	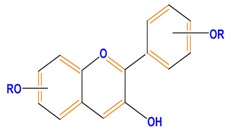	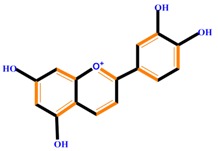	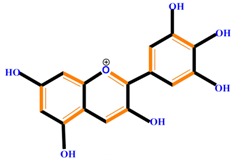	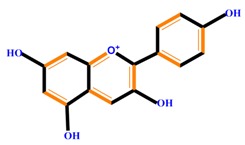
Lignans	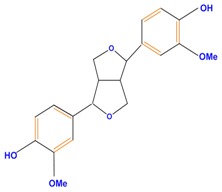	Pinoresinol		
		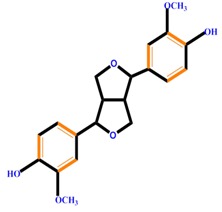		
Stilbenes	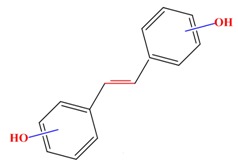	Resveratrol	Polydatin	
		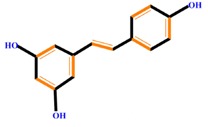	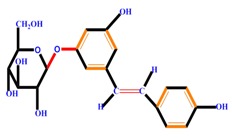	

**Table 2:** The most relevant PCs with anti-fungal activity.

Substance	Pathogen	Ref.
Oleuropein	*Phytophthora* spp.	[58]
Nobilietin	*Phoma tracheiphila*	[59]
Genistein	*Monilinia fructicola*	[60]
Biochanin	*Monilinia fructicola*	[60]
5,8-Dihydroxy-6,7-dimethoxyflavan	*Fusarium oxysporum*	[60]
Thymol	*Cryptococcus neoformans*, *Candida albicans*, *Rhyzopus* sp., *Aspergillus* sp.	[61,62]
Hispidulin	*Cladosporium sphaerospermum*	[59]
Flavone	*Aspergillus* sp.	[59]
Flavanone	*Aspergillus* sp.	[59]
Phloretin	*Venturiaina equalis*	[59]
Kaempferol	*Pyriclaria oryzae*	[59]
3-and 7-Hydroxyflavone	*Penicillium glabrum*	[59]
*p*-Coumaric acid	*Gelosporium perennas*	[59]
Rutin	*Fusarium oxysporum*	[59]
Vanillic acid	*Phytophthora infestans*	[59]
Salicylic Acid (SA)	*Eutypa lata, Penicillium expansum, Fusarium graminearum*	[63,64,65]
2,5-Dimethoxybenzoic acid	*Botrytis cinerea*	[59,66]
Catechol	*Colletotrichum circinans, Candida albicans*	[67,68]
Protocatehuic acid	*Colletotrichum circinans*	[69]
3,4-Dihydroxybenzaldehyde	*Gloesporium musarum*	[70]

**Table 3:** The most abundant allelochemical compounds and their mechanisms.

Allelochemical	Distribution	Mechanism	Ref.
*p*-Hydroxybenzoic acid	Leaves	Inhibiting enzymatic activity	[101]
*p*-Coumaric acid	Leaves	Growth inhibitor	[101]
Quercetin	Leaves, Root, Bark	Anti-insect (*Aphis* *craccivora* Koch)	[59]
2,4-Dihydroxy-1,4(2*H*) benzoxazin-3-one	Leaves, Root, Bark	Various actions	[59]
(−)-Catechin	Root	Inducing stress responses	[101]
Sorgoleone	Root	Photosystem II inhibitor, hydroxyphenyl pyruvate dioxygenase inhibitor	[101]
Phenolic acid	Root	Inhibiting seedling growth	[102]
Flavonoids	Root	Inhibiting seedling growth	[102]
SA	Root	Release of other allelochemicals	[103]
Cinnamic acid	Leaves	Inhibiting chlorophyll biosynthesis	[104]
Ferulic acid	Leaves, Root	Inhibiting of seed germination	[104]

**Table 4:** Anticancer activity of the most relevant PCs.

Compound	Mechanism	Type of Cancer	Ref.
Sophoranone	Inhibits cell growth, induces apoptosis	Human stomach cancer MKN7 cells, human leukemia U937 cells	[16,22,78]
Kaempferol 3-*O*-rutinoside	Anti-inflammatory	Gastric cancer	[22]
Kaempferol	Anti-inflammatory, induces apoptosis	Gastric cancer, prostate cancer, thyroid cancer (ARO, NPA, WRO cells)	[109]
Isoflavonoids (general)	Induces apoptosis	Breast cancer lines, lung cancer lines,	[22]
Nobiletin	Cell cycle arrest (G1 phase), inhibits angiogenic differentiation by Vascular endothelial growth factor (VEGF) and fibroblast growth factor (FGF), down-regulation of ERK1/2 and c-Jun N-terminal kinases (c-JNK), induces caspase pathway	Breast cancer cell lines	[110,111]
Quercetin	Inhibits cancer metastasis, inhibits MAPK phosphorylation, induces differentiation of HL-60 cells into granulocytes and monocytes	Gastric cancer, lung cancer (SK-LU1, SW900, H441, H661, haGo-K-1, A549 cells)	[112]
Chalcones	Inhibits cell growth	B16 mouse melanoma	[22,113]
Apigenin	Inhibits cancer metastasis, inhibits MAPK phosphorylation, induces apoptosis, induces differentiation of HL-60 cells into granulocytes and monocytes	Leukemia (HL-60, K562, Jurkat cells)	[114]
Flavone	Inhibits proliferation, induces apoptosis	Colon cancer (Caco-2, HT-29, IEC-6, HCT-15 cells)	[22,115]
Genistein	Inhibits proliferation, induces apoptosis	Prostate cancer (LNCaP, PC3, DU145 cells)	[116]
Daidzein	Inhibits proliferation, induces apoptosis	Breast cancer (MCF-7 cells)	[117]
Courcumin	Inhibits proliferation, induces apoptosis	Oral cancer (HSC-2, HSG, SCC-25 cells)	[112]
Catechin	Inhibits tumor-invasive activity, inhibits cell shedding, hepatocyte growth factor signaling, cell arrest in S phase, modulates NO signaling, induces killer caspases, inhibits NF-κB signaling	Same effect as genistein	[112]
Flavopiridol	Inhibits CDKs, induces cell cycle arrest during G_1_ or G_2_/M, induces apoptosis	Prostate, colon, and gastric cancers	[118]
Luteolin	Induces differentiation of HL-60 cells into granulocytes and monocytes	Colon cancer cells	[118,119]
Hesperetin	Represses CDK2, CDK4, and cyclin D, Induces p21 and p27 expression, blocks cell cycle in G_1_ phase , promotes apoptosis, suppresses proliferation, increases expression of caspase-3, caspase-8, caspase-9, p53, Bax, Fas	Liver cancer (HepG2 cells), cervical cancer (SiHa cells), leukemia (NALM-6 cells), breast cancer (MCF-7 cells)	[118]
5HTMF	Induces G_0_/G_1_ arrest, changes p21and p53 status	Colon cancer cells	[118]
Tangeretin	Induces caspase-3 activity, Cell cycle arrest (inhibit G_1_ phase), suppresses proliferation, inhibits cancer metastasis, Scavenging of ROS	Colon cancer cells, liver cancer (HepG2 cell), cervical cancer (SiHa cell)	[118]
Naringenin	Blocks cells in the G_0_/G_1_ and G_2_/M phases, induces metastasis, decreased ROS generation, induces TNF-α	Liver cancer (HepG2 cell), cervical cancer (SiHa cell)	[107,120,121]
Sinensetin	Antiangiogenesis, blocks G_0_/G_1_ phase, regulates expression of angiogenesis genes *flt1*, *kdrl*, and *hras*	General anticancer substances	[107]
Anthocyanins	Reduces inflammatory and tumor initiation, suppresses angiogenesis, minimizes cancer-induced DNA damage (in animal disease model)	General anticancer substances	[122]
Flavonols	Direct cellular proliferation inhibitor	Leukemia and pancreatic, breast, cervical, prostate, uterine, and urinary tract cancers.	[22]
Caffeoylquinic acids	Antioxidant activity	Limit LDL oxidation, general effect on cancer cell lines	[22]
Isoflavonoids	General protective activity	breast and prostate cancers	[123]
Resveratrol		Skin cancer, tumors of the gastrointestinal tract	[124]

CDK: cyclin-dependent kinase; LDL: low-density lipoprotein; MAPK: mitogen-activated protein kinase; NO: nitric oxide; TNF-α: tumor necrosis factor-α; ERK: extracellular signal-regulated kinase; NPA: UCLA NPA-87-1.

**Table 5:** Roles of the most relevant PCs in plant growth.

PCs	Roles during Plant Growth	Ref.
*p*-Coumaric acid	Cell wall development, seed germination, and dormancy	[129]
SA	Effect on accumulation of ABA and IAA, regulation of growth, ion uptake, photosynthetic performance, membrane permeability, response to drought, salt stress, heavy metals, and multiple-stress tolerance.	[132]
Ferulic acid	Cell wall development, Allelopathy (germination inhibitors), effect on accumulation of ABA, IAA, response to abiotic stress	[129]
Caffeic acid	Antioxidant, light absorption	[136]
Cinnamic acid	Effect on accumulation of ABA, IAA, response to abiotic stress	[136]
Tyramine	Reduce cell count and dwarfing	[129]
Hydroxycinnamic acids	Decrease of lignification during abiotic stress, response to water tension, seed germination, and dormancy	[129]
Hydroxycinnamoylquinic acids	Response to water stress	[129]
Hydroxycinnamic acid glucosides	Response to water stress	[129]
SA glucoside	Response to water stress	[137]
Conjugated flavonols (with disaccharides)	Response to water stress	[129]
Caffeoylputrescine	Response to water stress	[129]
Isoflavonoids	Phytoalexins	[129]
Tannins	Defensive properties by binding to proteins, Tolerant to heavy metal	[138]
Flavons and Flavonols	Plant growth development by absorb light, protect cells from excessive UV radiation, legume nodulations and nitrogen-fixing, membrane stabilizer during stresses	[138]
Anthocyanin	Attracting pollinators	[139]
Flavonoids	Flower pigmentation, UV-protection, plant defense, legume nodulations, membranes stabilizer during stress, scavenging of reactive species (ROS, H_2_O_2_, etc.)	[140]
Lignin	Xylogenesis, defensive response to pathogen, cell wall formation	[139]
Apigenin	Compete with IAA and inhibit polar auxin transport	[59]
Gallic acid 4-*O*-(β-d-glucopyranosyl-6′-sulfate)	Control of nyctinastic movement in leaves	[59]
Gentisic acid 5-*O*-β-d-glucopyranoside	Control of nyctinastic movement in leaves	[59]
Kaempferol	Compete with IAA and inhibit polar auxin transport	[59]
Ascorbic acid	Antioxidant activity and protection of cells	[138]
Isoflavone	Response to environmental tensions	[141]
*o*-Dihydroxy phenolics	Anti-herbivore activity	[138]
Simple Phenolics	Plant–environment interactions and allelopathy	[139]
Phenylpropanoid	Lignin biosynthesis	[139]
Monohydroxy B-ring Flavonoids	Decompose IAA hormone, prevent of IAA transport by binding to NPA	[59]
Dihydroxy B-ring Flavonoids	Preventers of the IAA action, preventers of IAA transport by binding to NPA	[59]

ABA: abscisic acid; IAA: indoleacetic acid; NPA: nephthylphtalamic acid; ROS: reactive oxygen species.

**Table 6:** Use of PCs in plant tissue culture.

Compound	Activity	Ref.
Phloroglucinol (1,3,5-trihydroxybenzene)	Increase growth and axillary shoot generation, prevention of vitrification, increase somatic embryogenesis, control of hyperhydricity in lignification	[132]
Phloroglucinol + NAA	Higher levels of somatic embryogenesis	[135]
Phloroglucinol + BA	Improve number of shoots	[134]
Phloroglucinol + any cytokinins	100% regeneration	[135]
Phloretic acid	Increase shoot and root	[135]
Phloroglucinol + IAA	Increase rooting	[142]
Chlorogenic acid	Stimulate callus growth	[143]
Glycoside phloridzin	Same effect as phloroglucinol	[132]
Quinone	Negative effect on cell growth (by death/necrosis)	[144]

## Abbreviations

PCs	Phenolic compounds
EGFR	Epidermal Growth Factor Receptor
EGF	Epidermal Growth Factor
Her2/neu	Human Epidermal Growth Factor Receptor 2
HR	Hypersensitive response
PCD	Programed cell death
PAL	Phenylalanine ammonia-lyase
PPO	Polyphenol oxidase
POH	Polyphenolic antioxidants
POX	Peroxidase
ROS	Reactive Oxygen Species
RNS	Reactive Nitrogen Species
H_2_O_2_	Hydrogen Peroxide
OH	Hydroxyl Radicals
SA	Salicylic acid
NPA	Nephthylphtalamic acid
IAA	Indoleacetic acid
ABA	Abscisic acid
PVP	Polyvinylpyrrolidone
NAA	Naphthaleneacetic acid
MAPK	mitogen-activated protein kinase
CDKs	Cyclin-dependent kinases
5HTMF	5-hydroxy-6,7,8,3′,4′-pentamethoxyflavone
PO•	Phenoxyl radical
ABCBs	ATP-binding cassette subfamily B
